# Infantile Pain Episodes Associated with Novel Nav1.9 Mutations in Familial Episodic Pain Syndrome in Japanese Families

**DOI:** 10.1371/journal.pone.0154827

**Published:** 2016-05-25

**Authors:** Hiroko Okuda, Atsuko Noguchi, Hatasu Kobayashi, Daiki Kondo, Kouji H. Harada, Shohab Youssefian, Hirotomo Shioi, Risako Kabata, Yuki Domon, Kazufumi Kubota, Yutaka Kitano, Yasunori Takayama, Toshiaki Hitomi, Kousaku Ohno, Yoshiaki Saito, Takeshi Asano, Makoto Tominaga, Tsutomu Takahashi, Akio Koizumi

**Affiliations:** 1 Department of Health and Environmental Sciences, Graduate School of Medicine, Kyoto University, Kyoto, Japan; 2 Department of Pediatrics, Akita University School of Medicine, Akita, Japan; 3 Laboratory of Molecular Biosciences, Graduate School of Medicine, Kyoto University, Kyoto, Japan; 4 Biological Research Laboratories, Daiichi Sankyo Co., Ltd., Shinagawa-ku, Tokyo, Japan; 5 Okazaki Institute for Integrative Bioscience, National Institute for Physiological Sciences, National Institutes of Natural Sciences, Aichi, Japan; 6 Department of Preventive Medicine, St. Marianna University School of Medicine, Kanagawa, Japan; 7 Department of Pediatrics, Sanin Rosai Hospital, Tottori, Japan; 8 Division of Child Neurology, Institute of Neurological Sciences, Faculty of Medicine, Tottori University, Tottori, Japan; 9 Department of Pediatrics, Nippon Medical School Chiba Hokusoh Hospital, Chiba, Japan; Southern Illinois University School of Medicine, UNITED STATES

## Abstract

Painful peripheral neuropathy has been correlated with various voltage-gated sodium channel mutations in sensory neurons. Recently Nav1.9, a voltage-gated sodium channel subtype, has been established as a genetic influence for certain peripheral pain syndromes. In this study, we performed a genetic study in six unrelated multigenerational Japanese families with episodic pain syndrome. Affected participants (*n* = 23) were characterized by infantile recurrent pain episodes with spontaneous mitigation around adolescence. This unique phenotype was inherited in an autosomal-dominant mode. Linkage analysis was performed for two families with 12 affected and nine unaffected members, and a single locus was identified on 3p22 (LOD score 4.32). Exome analysis (*n* = 14) was performed for affected and unaffected members in these two families and an additional family. Two missense variants were identified: R222H and R222S in *SCN11A*. Next, we generated a knock-in mouse model harboring one of the mutations (R222S). Behavioral tests (Hargreaves test and cold plate test) using R222S and wild-type C57BL/6 (WT) mice, young (8–9 weeks old; *n* = 10–12 for each group) and mature (36–38 weeks old; *n* = 5–6 for each group), showed that R222S mice were significantly (*p* < 0.05) more hypersensitive to hot and cold stimuli than WT mice. Electrophysiological studies using dorsal root ganglion neurons from 8–9-week-old mice showed no significant difference in resting membrane potential, but input impedance and firing frequency of evoked action potentials were significantly increased in R222S mice compared with WT mice. However, there was no significant difference among Nav1.9 (WT, R222S, and R222H)-overexpressing ND7/23 cell lines. These results suggest that our novel mutation is a gain-of-function mutation that causes infantile familial episodic pain. The mouse model developed here will be useful for drug screening for familial episodic pain syndrome associated with *SCN11A* mutations.

## Introduction

The recent identification of genetic variants causing pain-related disorders has created a new window into the etiology of hereditary pain. Voltage-gated sodium ion channels have been intensively studied, with a broad spectrum of new findings uncovered for peripheral neuropathy. Among the nine subtypes (Nav1.1–Nav1.9) encoded by the genes *SCN1A*–*SCN5A* and *SCN8A*–*SCN11A*, three subtypes (Nav1.7, Nav1.8, and Nav1.9) are strongly expressed in sensory neurons, and have been associated with human pain disorders. Mutations in *SCN9A* cause a spectrum of clinical phenotypes ranging from congenital insensitivity to pain [1], as well as chronic pain syndromes such as paroxysmal extreme pain disorder [2,3], inherited erythromelalgia [4,5], and small fiber neuropathy [6]. In contrast, Nav1.8 is associated with phenotypes ranging from painful symptoms [7] to Brugada syndrome [8].

Nav1.9, which is encoded by *SCN11A*, is a tetrodotoxin (TTX)-resistant (TTXr) channel primarily expressed in nociceptors. Nav1.9 channels exhibit hyperpolarized voltage-dependent activation close to the resting membrane potential (RMP), with slow inactivation leading to a persistent current [9]. Hence, Nav1.9 is thought to be an important regulator of membrane excitability. Recently Nav1.9 was identified as the genetic background for some peripheral pain syndromes. In a German family with an unusual syndrome involving loss of pain sensation, a *de novo* missense *SCN11A* mutation (p.L811P; we have abbreviated the prefix “p” in this paper) was found [10]. In another screening of *SCN11A* in 58 independent individuals with early onset severe sensory loss, a Swedish male was identified as a patient with exactly with the same heterozygous mutation (L811P), again occurring as a *de novo* event. This Nav1.9 variant was examined electrophysiologically in *Scn11a*^+/L799P^ knock-in mice (L799P being the mouse ortholog), and exhibited excess sodium ion influx at rest with subsequent cell depolarization [10].

In contrast, two large multigenerational Chinese families with autosomal-dominant episodic chronic pain and two types of gain-of-function *SCN11A* mutations (R225C and A808G) were identified by genome-wide linkage analysis combined with whole-exome sequencing [11]. Overexpression of these mutants in mouse dorsal root ganglion (DRG) cells induced hyperexcitability [11].

Recently, *SCN9A*, *SCN10A*, and *SCN11A* screening efforts in 393 patients with painful peripheral neuropathy revealed causative *SCN11A* mutations (L1158P and I381T) [12]. Onset of pain in these affected individuals was characteristically in adulthood, and therefore different from the infantile period in the Chinese cases with *SCN11A* mutations. Further, the L1158P and I381T mutations were often uniquely accompanied by burning sensations, and sometimes autonomic dysfunction such as dry eyes, diarrhea, and urinary problems [12].

Collectively, Nav1.9 channelopathy is associated with diverse clinical phenotypes: painful and painless peripheral neuropathy, as well as autonomic symptoms. Further studies are needed to establish phenotype-genotype correlations in Nav1.9 channelopathy.

Here, we report two novel Nav1.9 variants (R222H and R222S) in patients of six unrelated familial episodic pain syndrome. Furthermore, we generated a mouse model harboring one of the mutations, R222S, and evaluated the model using a battery of behavioral tests. We succeeded in partially recapitulating the clinical phenotype. Finally, we examined the electrophysiological mechanisms of the Nav1.9 variant in DRG neurons and Nav1.9-overexpressing ND7/23 cell lines, and found that the R222S mutation was associated with increased firing probability in DRG neurons.

## Materials and Methods

### Ethical statements

A part of the human study was approved by the Institutional Review Board and Ethics Committee of Kyoto University School of Medicine (approval no. G501; approval date 2 Aug. 2012), and Japan and Akita University Graduate School of Medicine (approval no. 960; 26 Sep. 2012), Japan. Written informed consent was obtained from all subjects and the parents of children and adolescents before participation.

Animal studies including animal care and all experimental procedures were in accordance with the Animal Welfare Guidelines of Kyoto University or the in-house guidelines of the Institutional Animal Care and Use Committee of Daiichi Sankyo Co., Ltd. Animal experiment protocols were reviewed and approved by the Animal Care, Use and Ethics Committee at Kyoto University (approval no. MedKyo15074; 27 Mar. 2015) and the Institutional Animal Care and Use Committee of Daiichi Sankyo Co., Ltd. (approval no. A1500635, 26 Mar. 2015 [8–9-week-old mice experiment]; A1502171, 16 Oct. 2015 [36–38-week-old mice experiment]).

### Pedigrees and genomic DNA isolation

**Fig 1:**
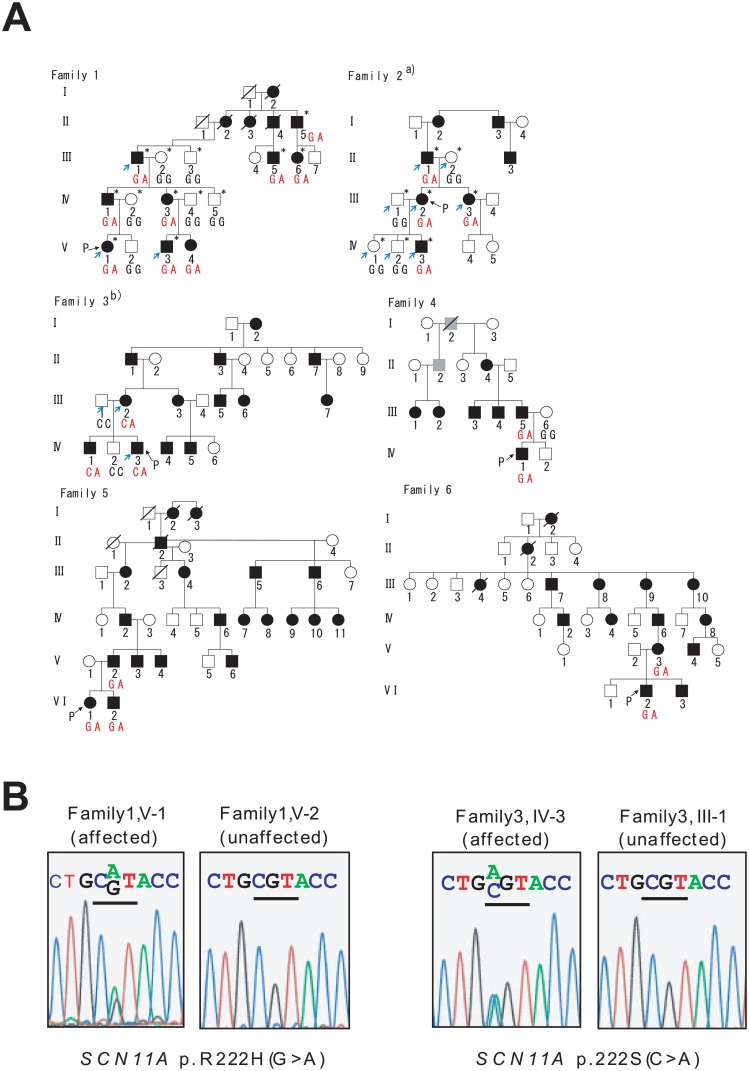
Pedigrees of six Japanese familial episodic pain syndrome in Japanese families. (A) Some ^a)^Family 2 and ^b)^Family 3 members have been reported previously [13, 14]. Black and white symbols indicate affected and unaffected individuals, respectively. Gray symbols indicate individuals with unknown phenotypic status. Squares and circles indicate males and females, respectively. Slashes indicate deceased individuals. “P” indicates probands. Blue arrows indicate exome sequenced individuals. * indicates linkage analysis performed individuals. The genotype of *SCN11A* p.R222H (Family 1, 2, 4, 5 and 6) or *SCN11A* p.R222S (Family 3) for each individual is illustrated. (B) Sequence chromatography of the identified *SCN11A* mutations.

Six unrelated families were examined in this study (Fig 1A). Each family included a number of affected and unaffected members. Peripheral blood was collected from 23 affected members and 13 unaffected members from these families. Genomic DNA was extracted from whole blood samples using the QIAamp DNA Blood Mini Kit (Qiagen, Hilden, Germany).

### Linkage analysis

Eight affected members (II-5, III-1, III-5, III-6, IV-1, IV-3, V-1, and V-3) and five unaffected members (III-2, III-3, IV-2, IV-4, and IV-5) in Family 1 and four affected members (II-1, III-2, III-3, and IV-3) and four unaffected members (II-2, III-1, IV-1, and IV-2) in Family 2 (Fig 1A) were analyzed by genome-wide linkage analysis. Microsatellite polymorphisms (382 markers, 10 cM apart) covering 22 autosomes were genotyped using ABI Prism Linkage Mapping Set (Version 2; Applied Biosystems, Foster City, CA, USA) as well as additional SNP markers. Parametric linkage analysis was performed using 386 genetic markers by GeneHunter (Ver. 2.1_r6) [15] under an autosomal-dominant mode of inheritance. Disease allele frequency was set at 10^−5^ and phenocopy frequency at 10^−6^. For individual alleles, population allele frequencies were assigned equal proportions. Haplotypes in the families were estimated using GeneHunter [15].

### Whole-exome sequencing

Whole-exome sequencing was performed for 14 subjects: nine affected and five unaffected members:, specifically, three affected members in Family 1 (III-1, V-1, and V-3), four affected members (II-1, III-2, III-3, and IV-3) and four unaffected members (II-2, III-1, IV-1, and IV-2) in Family 2, and two affected members (III-2 and IV-3) and one unaffected member (III-1) in Family 3 (Fig 1A). Whole-exome sequencing was performed at Riken Genesis Co., Ltd (Kanagawa, Japan). The target region (exonic regions and flanking intronic regions) was captured using the SureSelect Human All Exon V4+UTR Kit (Agilent Technologies, Santa Clara, CA, USA), and sequencing performed using the Illumina HiSeq 2000 platform (Illumina Inc., San Diego, CA, USA). Sequence reads were mapped to the reference human genome (UCSC Genome Browser hg19, http://hgdownload.cse.ucsc.edu/goldenPath/gh19/chromosomes/) using Burrows-Wheeler Aligner software (Ver. 0.6.2) (http://bio-bwa.sourceforge.net/index.shtml). Single-nucleotide variants and small insertion/deletions were detected using Genome Analysis Toolkit software (Ver. 1.6–13) (https://www.broadinstitute.org/gatk/). A series of filters were used to identify candidate variants as follows: (1) non-synonymous variants (missense, nonsense, read through, splice site variants, and indels), (2) high read depth (≥ 8), (3) not registered in dbSNP135 database (ftp://ftp.ncbi.nlm.nih.gov/snp/organisms/human_9606/ASN1_flat), (4) less common (minor allele frequency (MAF) < 0.01) in Japanese patients from the 1000 Genomes database (Oct 2011) (ftp://ftp.1000genomes.ebi.ac.uk/vol1/ftp/release/20110521/), (5) heterozygote variant in all affected members and not present in unaffected members of each family, (6) located in a linkage region, and (7) variants in the same gene among the three families. For confirmation of the mutations, the *SCN11A* gene coding region was sequenced by the Sanger method using primers described in S1 Table.

### Restriction fragment length polymorphism method

Genotyping of R222H and R222S in the general population was performed by restriction fragment length polymorphism (RFLP) using CviQI and DdeI. The primers and restriction enzymes used are described in S2 Table.

### Nav1.9 knock-in mouse

In mouse, R222S alteration is the allelic ortholog of the human R222S mutation, and was introduced into the mouse *Scn11a* locus using the CRISPR/CAS9 system by TransGenic Inc. (Kumamoto, Japan). Single guide RNAs (sgRNAs) targeting the region around the mouse *Scn11a* R222 locus were designed using the Optimized CRISPR Design web tool (http://crispr.mit.edu/) [16]. To avoid off-target effects, two sgRNAs were designed. Both oligonucleotide DNAs coding sgRNAs (S3 Table) were synthesized, annealed, and cloned into the pX330-U6-Chimeric_BB-CBh-hSpCas9 plasmid [17] obtained from Addgene (Addgene plasmid # 42230). Single-strand donor oligonucleotide DNA (donor oligoDNA) harboring a nucleotide variant that introduces the R222S amino acid change was synthesized (Integrated DNA Technologies, Coralville, IA, USA) (S3 Table). Cas9+sgRNA vector and donor oligoDNA were microinjected into fertilized C57BL/6 mouse eggs to generate *Scn11a*^+/R222S^ mice. The nucleotide change in genomic DNA that corresponds to *Scn11a* R222S was confirmed in offspring by direct sequencing using the primers described in S3 Table. Further genotyping was performed using TaqMan SNP genotyping assays (Applied Biosystems).

### Behavioral experiments

Male knock-in (R222S mouse) and wild-type C57BL/6 mice (WT) were transferred from Kyoto University to Daiichi Sankyo Co., Ltd. Mice were housed in an air-conditioned room with controlled temperature (23 ± 2°C) and humidity (55 ± 10%) and a 12-h light/dark cycle (light from 7:00 to 19:00). They were fed and given water *ad libitum*. In the following behavioral experiments, the mice were used at 8–9 or 36–38 weeks old.

Irwin’s test: multiple neurobehavioral signs (a total of 45 observation items including awareness, mood, motor activity, CNS excitation, posture, motor coordination, muscle tone, reflexes, and autonomic neurological signs and miscellaneous symptoms) were observed according to the method of Irwin [18].

Locomotor activity: locomotor activity was measured using SCANET MV-40 (MELQEST, Toyama, Japan), a device that uses infrared sensors to detect movement of an animal placed individually in a transparent plastic cage (20 cm × 20 cm × 20 cm). Once the animal was placed into the cage, detection of movement was started. Total horizontal movement was recorded for 30 min.

Von Frey test: sensitivity to a mechanical stimulus was assessed using von Frey filaments (Aesthesio^®^, DanMic Global, LLC, San Jose, CA, USA). Each animal was placed individually into a transparent plastic cage (8 cm × 7 cm × 8 cm) with a wire-mesh floor to allow insertion of the filament from below. The filament was placed against the plantar surface of the left hindpaw and acclimatized for not less than 30 min before testing. A 50% threshold, which indicates the filament force at which an animal reacts in 50% of presentations, was determined using a previously reported method [19]. Eight filaments with approximately equal logarithmic incremental forces (0.02, 0.04, 0.07, 0.16, 0.4, 0.6, 1.0, and 1.4 g) were used.

Hargreaves test: sensitivity to a thermal stimulus was assessed using the PAW THERMAL STIMULATOR (UCSD, San Diego, CA, USA). Each animal was placed individually in a transparent plastic chamber (8 cm × 8 cm × 18 cm) with a glass floor, and acclimatized for not less than 30 min before testing. A radiant light heat source located under the floor was focused onto the plantar surface of the left hindpaw, and paw withdrawal latencies recorded. Latencies were measured in triplicate for the left hindpaw of each animal, and the mean used for analysis. A 20 sec cut-off time was employed to avoid tissue damage.

Cold plate test: sensitivity to a cold stimulus was assessed using the MK-350HC Hot/Cold Plate Analgesia Meter (Muromachi Kikai Co., Ltd., Tokyo, Japan). Each animal was placed individually in a transparent plastic cylinder cage (8 cm φ × 20 cm) with a cold plate floor at 10°C. The total number of nociceptive behaviors (e.g., lifting and shaking of paws and jumping) was counted for 3 min.

### Nav1.9-overexpressing ND7/23 cells

Nav1.9-overexpressing ND7/23 cells, mouse neuroblastoma cells and rat DRG neurons hybrid cell line, were produced using the *PiggyBac* transposon system, as previously reported [20]. Briefly, cDNA comprising human Nav1.9 (WT, R222H mutant, and R222S mutant) coding region (NM_014139.2) fused with 3xFLAG at the C terminus was synthesized by DNA2.0 Inc. (Menlo Park, CA, USA), and cloned into a transposon vector (pJ508) containing a blasticidin resistance gene (DNA2.0 Inc.). Nav1.9-3xFLAG transposon vectors were then transfected into ND7/23 cells using FuGene6 (Promega, Madison, WI, USA). Cells stably expressing Nav1.9-3xFLAG were selected in 5 μg/ml blasticidin (Invitrogen, Carlsbad, CA, USA), isolated from individual cell colonies using a cloning disk, and expanded. Nav1.9-3xFLAG expression was confirmed by immunocytochemistry and real-time quantitative PCR.

### Immunocytochemistry

Nav1.9-overexpressing ND7/23 cells cultured at 28°C for 24 h were fixed in methanol, permeabilized with acetone, and blocked in 3% BSA. The cells were then incubated with a mouse monoclonal anti-FLAG antibody (M2 clone; Sigma-Aldrich, St. Louis, MO, USA), followed by Alexa Fluor 594-conjugated anti-mouse IgG (Thermo Fisher Scientific, Waltham, MA, USA). Fluorescent signals were examined using a TCS SP8 confocal laser microscope (Leica, Heidelberg, Germany).

### Real-time quantitative PCR

cDNA was obtained from Nav1.9-overexpressing ND7/23 cells using the High-Capacity cDNA Reverse Transcription Kit (Applied Biosystems). Real-time quantitative PCR for human *SCN11A* cDNA was performed using the primers described in S4 Table. Target cDNA expression levels were normalized to the corresponding *Ppia* expression levels.

### Isolation of DRG neurons

DRG neurons were isolated, as previously reported [21], from L4 to L6 sections of 6–8-week-old WT and R222S mice. Briefly, mice were anesthetized using sevoflurane and then perfused in artificial cerebrospinal fluid (aCSF; [in mM] 124 NaCl, 5 KCl, 1.2 KH_2_PO_4_, 1.3 MgSO_4_, 2.4 CaCl_2_, 10 glucose, and 24 NaHCO_3_). DRG neurons were extracted from the spinal cord and isolated with collagenase XI (Sigma-Aldrich) in incubation medium containing Earle’s balanced salt solution (Sigma-Aldrich), in a 25 min incubation at 37°C. After collagenase digestion, DRG cells were dispersed using fire-polished Pasteur pipettes and centrifuged three times. Isolated DRG neurons were resuspended in aCSF and plated onto non-coated 12 mm φ coverslips.

### Electrophysiology

#### Nav1.9-overexpressing ND7/23 cells

Nav1.9-overexpressing ND7/23 cells (WT and mutant [R222H, R222S]) and control cells (ND7/23 cells without Nav1.9 transfection) were inoculated onto 12 mm φ glass coverslips, and incubated at 28°C with 5% CO_2_ for 16–24 h before patch clamp recording. Currents were recorded using an Axopatch 200B amplifier (Molecular Devices, Sunnyvale, CA, USA), low-pass filtered at 5 kHz, and digitized using a Digidata 1440A (Axon Instruments, Union city, CA, USA). The pipette solution contained (in mM): 10 NaF, 110 CsF, 20 CaCl_2_, 2 EGTA, and 10 HEPES (pH 7.3 with KOH). The bath solution contained (in mM): 130 NaCl, 5 KCl, 1 MgCl_2_, 2 CaCl_2_, 10 glucose, and 10 HEPES (pH 7.4 with NaOH). To select cells with overexpressed sodium channels and block TTX-sensitive (TTXs) channels, blasticidin (50 μg/ml) and TTX (3 μM; Wako Pure Chemical Industries, Osaka, Japan) were added to the bath solution. Patch pipettes were fabricated from thin-walled borosilicate glass capillaries (King Precision Glass, Inc., Claremont, CA, USA), and had a resistance of ~3.5 MΩ [22]. Series resistance was monitored during the recording session, and data were rejected if their value changed by > 20%. Leak currents were subtracted using electrical subtraction protocols (p/4). Data were acquired by pCLAMP 10 (Axon Instruments). Voltage-dependent currents were acquired after achieving more than 5 min of whole-cell recording conditions. To record the current/voltage (I/V) relationship of TTXr, cells were held at −100 mV and step pulses applied from −120–30 mV for 100 ms in 10-mV increments. We did not include data for WT, R222S and R222H with the peak currents more than −10 pA, which corresponds with the peak amplitude of the control cells (See results). Current traces were filtered with a low-pass Bessel setting of 10 kHz. Activation curves were obtained above the step pulse and fitted to the Boltzmann function in the form of G = G_max_ /{1+exp[(V_1/2_ − V)/k]}, where G_max_ is the maximum conductance, V_1/2_ is the mid-point of the activation curve, and k is the slope factor. All data were obtained at 28°C.

#### Isolated DRG neurons

Electrophysiological data from isolated DRG neurons were collected from small diameter (< 25 μm) cells from WT and knock-in mice. Data were obtained at 23–25°C within 8 h after isolation using an EPC-9 amplifier (HEKA Elektronik, Lambrecht, Germany). Patch pipettes were fabricated from thin-walled borosilicate glass capillaries (GC150TF-10, Harvard Apparatus, MA, USA) and had a resistance of 4–6 MΩ [23, 24]. Patch pipette tips were fire-polished before use. Electrode capacitance was compensated electrically, and series resistance was < 13 MΩ. Data were collected in cells were which required smaller current than -30pA to hold the membrane at -60 mV. A previous study found that mouse DRG neurons initially up-regulate I(NaN) after cell rupture [25], therefore to avoid dispersion of this effect on action potential (AP) firing, current-clamp recordings were obtained after achieving more than 5 min of whole-cell recording conditions. The pipette solution contained (in mM): 67 KCl, 65 K-gluconate, 1 MgCl_2_, 5 EGTA, 4 ATP-Mg, 1 GTP-Na_2_, and 10 HEPES (pH 7.3 with KOH). The bath solution for isolated DRG neurons contained (in mM): 130 NaCl, 5 KCl, 1 MgCl_2_, 2 CaCl_2_, 10 glucose, and 10 HEPES (pH 7.4 with NaOH). TTX (1 μM) was added to the bath solution to block TTXs sodium channels.

RMP was measured when the membrane potential was stabilized over 5 min after the whole-cell recording configuration was achieved. RMP was measured immediately before current injection in every trial. The following parameters of the first AP were measured: amplitude, 50% AP width, and maximum rate of rise/fall of AP. To evaluate input impedance, the voltage response amplitude was measured at a current injection of 10 pA. AP frequency was calculated from the AP number during step current injections (500 ms) from 10 to 210 pA in 25 pA increments. The current injection was limited to 210 pA because firing was not observed with currents larger than 210 pA owing to sodium channel inactivation. Cells that did not generate APs [26] or had only one AP in response to a 500-ms current stimulus in all step pulses were excluded from analysis. All data were acquired using Patchmaster (HEKA Elektronik). Electrophysiological data of isolated DRG neurons were analyzed using Igor Pro (WaveMetrics Inc., Portland, OR, USA).

### Statistical analysis

Behavioral test and electrophysiological data were presented as mean ± standard error of the mean (S.E.M.). Statistical significance was determined by two-sided Student’s *t*-tests. All statistical analyses were performed using SAS software (Version 9.4; SAS Institute, Cary, NC, USA). Statistical significance was indicated by *p* < 0.05.

## Results

### Phenotypic characterization

The clinical features of the six pedigrees examined in this study (Fig 1A) are described below.

#### Family 1

The proband (V-1) is a 4-year-old girl born to nonconsanguineous parents after an uneventful delivery. Recurrent, unexpected crying and unpleasantness were recognized during her infancy. Around the age of 2 years, the mother noticed this was due to paroxysmal limb pain. The patient grew up normally and developed without other problems. The pain was partially relieved with oral acetaminophen, but was persistent, leading to referral to our hospital at the age of 4 years.

The characteristics of her limb pain were that it began abruptly and continued for 15–30 min, but occasionally for half or even a whole day. Typically the pain was induced by fatigue or weather changes, specifically atmospheric depression leading to cold weather, rain, and snow. The paroxysmal pain episodes occurred approximately 10 and 20 days a month during summer and winter, respectively. The patient is afebrile during an episode and has a cold sensation on the affected limb, being one or two elbows, wrists, knees, and ankles, without any other symptoms e.g., redness, rashes, swelling, and joint destruction. Physical examination did not reveal any abnormalities. Laboratory assessment did not show any suggestion of collagen diseases, autoimmune diseases, or other disorders. Her laboratory data was normal, as follows: peripheral blood count, coagulation system, erythrocyte sedimentation rate, C-reactive protein, ferritin, serum amyloid A protein, soluble interleukin receptor 2, IgD, complement levels, anti-nuclear and anti-cardiolipin antibodies, rheumatoid factor, anti-CCP antibody, serum lactate and pyruvate levels, amino acid analysis, and leukocyte alpha-galactosidase activity.

The father of the proband (IV-1), a 41-year-old man, showed similar symptoms during childhood. However, his paroxysmal pain episodes gradually decreased from around the age of 15 years, and now occur only once or twice a year. He sometimes has a throbbing headache, but has not yet been assessed in a hospital.

The other patients (IV-3, III-1, III-5, III-6, and II-5) all showed a similar clinical course. Their paroxysmal pain episodes decreased during their adolescence. This family is originally from Akita Prefecture.

#### Family 2

This family has previously been reported [13]. The proband (III-2), a 51-year-old woman, had paroxysmal and cyclic limb pain in her childhood. Although her paroxysmal pain episodes gradually decreased from around 15 years of age, intermittent episodes made her visit a physician when she was 36 years old. She noticed her son had the same symptoms (IV-3). The limb pain affected her quality of life; e.g., she could not attend elementary school. From our investigation, we identified four males and three females in her family who all showed clinical features similar to the proband’s. Their limb pain is almost identical to that of Family 1. However, it lasts for a longer time in some individuals and repeats several times after an intermittent period of a few hours, resulting in a series of pain. Their limb pain characteristically precedes weather changes, in particular before low air pressure and bad weather; therefore, they can anticipate impending bad weather. Indeed, “weather forecaster” has become their nickname within the family. With regards age dependency, the paroxysmal pain episodes gradually decreased from around 15 years of age in all affected individuals. They have frequently used acetaminophen for pain relief. This family is originally from Chiba Prefecture.

#### Family 3

This family has previously been reported [14]. Briefly, eight males and five females are affected with similar limb pain as in the other families. The proband (IV-3), a 3-year-old boy, started recurrent episodes of unexplained crying at 6 months of age, which was recognized to be the result of limb pain. Besides the shared feature of limb pain, he occasionally had migraine episodes. Paroxysmal limb pain and migraine have not occurred simultaneously. This family is originally from Tottori Prefecture.

#### Families 4, 5, and 6

Four males and three females are recognized as affected with paroxysmal limb pain in Family 4. Ten males and 10 females are confirmed to have paroxysmal limb pain in Family 5. Headache (not yet diagnosed as migraine) is another complaint in an individual of this family (V-2). Finally, six males and nine females had paroxysmal limb pain during their infancy in Family 6. In these three families, age dependency was confirmed and the paroxysmal limb pain episodes decreased from around 15 years of age. These families are originally from Akita Prefecture.

In summary, the affected joints in frequency order are: knees, ankles, wrists, and elbows. Occasionally, the pain is localized to the forearms, brachium, palm, fingers, thigh, lower leg, and acrotarsium. The pain usually occurs on one side of the extremities, but occasionally on both sides. The pain typically lasts for 15–30 min and recurs several times a day. Moreover, it tends to be more frequent and severe at night than daytime. The patients had recurrent episodes of night-time crying and sleep disturbances in childhood. These episodes were usually present during the first few days of a series of painful days. The pain is often induced by fatigue and a prelude of bad weather. The patients feel cold in the pain region and get relief by warming the lesion. The paroxysmal pain episodes appear to start during infancy and gradually decrease from around 15 years of age. Finally, six affected individuals (family 2, IV-3; family 3, III-3, IV-3, IV-4; family 6, V-3, VI-2) complained of anorexia and diarrhea following cessation of a series of pain. These gastrointestinal manifestations are not observed in other mutation carriers or unaffected members, being not segregated by Nav1.9 mutation.

### Clinical characterization

Almost all patients had a cold sensation in the affected limb. Therefore, we evaluated body surface temperature by thermography in four patients during their pain attacks.

In a previous report [14], the surface of the affected limb had a lower temperature than the contralateral side. In Family 3, it was higher in the affected forearm at the end of the pain attack. We also examined body surface temperature in the other families (1, 2, 4, and 6), but no significant difference was detected between the affected and contralateral limbs.

Nerve conduction velocity of the right median, right ulnar, right peroneal, and right tibial nerves was individually examined in an individual from Family 1 (IV-1), and all showed normal findings. None of the affected subjects had shown a past electrocardiographic abnormality.

### Genetic analysis

**Fig 2:**
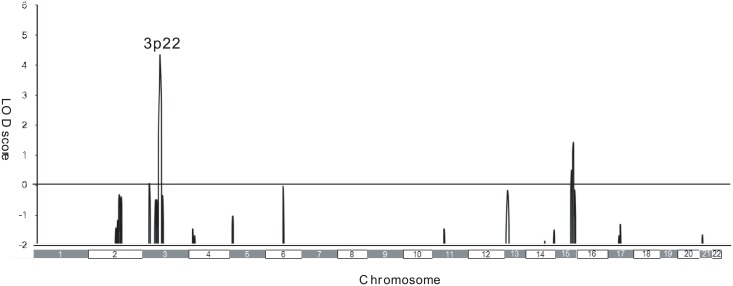
Genome-wide linkage analysis in two Japanese familial episodic pain syndrome families. Genome-wide linkage analysis was performed for eight affected and five unaffected members in Family 1, and four affected and four unaffected members in Family 2. Parametric linkage analysis was performed using 386 genetic markers (including 382 microsatellite genetic markers) that were 10 cM apart and covered 22 autosomes, as well as additional SNP markers. GeneHunter software was used.

To identify the causative genes, we used a combined approach of linkage analysis and exome sequencing. We first performed linkage analysis using Family 1 and 2, and identified a single locus on chromosome 3p22 with a LOD score of 4.32 (Fig 2), suggesting that the causative gene is located in this region.

**Fig 3:**
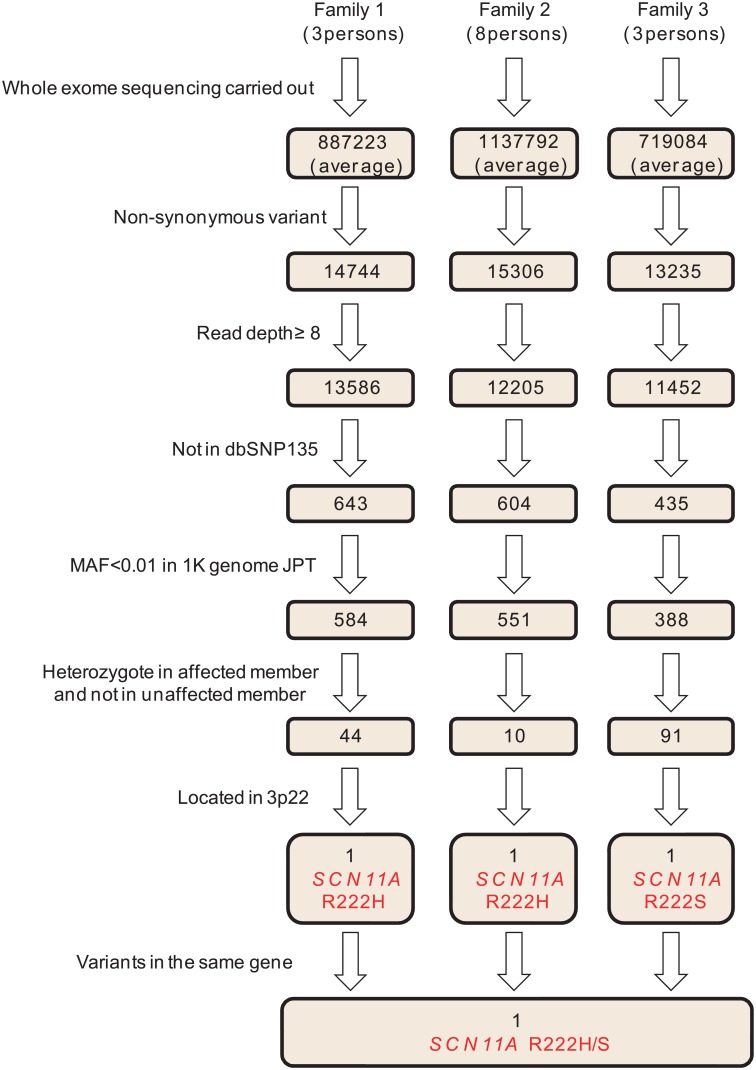
Exome analysis filtering process in the three Japanese familial episodic pain syndrome families. Exome analysis was performed for three affected members in Family 1, four affected and four unaffected members in Family 2, and two affected and one unaffected member in Family 3. Exome data was processed through seven filtering steps: (1) non-synonymous, (2) read depth ≥ 8, (3) not registered in dbSNP135, (4) MAF < 0.01 in Japanese patients from 1000 Genomes database, (5) heterozygote in affected members and not present in unaffected members, (6) located on 3p22 linkage region, and (7) variants in the same gene among all three families. Numbers in boxes represent the numbers of variants after each filtering step.

We next performed exome analysis using three families (Fig 1A). Whole-exome sequencing generated 9.3–14.7 billion bases from nine affected and five unaffected individuals. After mapping to the human reference genome (UCSC Genome Browserhg19), we obtained 6.8–12.0 Gb of targeted exome sequence suitable for mapping, with a mean sequencing depth between 64- and104-fold. The mean percentage of exomes covered a read depth of ≥ 8-fold, which was > 94.7%. Filtering of variants was performed, with a focus on less common, deleterious variants in coding sequence regions. We removed synonymous and low coverage (read depth < 8) variants, and filtered against two reference databases (dbSNP135 and 1000 Genomes). Given that the families show an autosomal-dominant inheritance pattern, we selected variants that were heterozygote in affected members and not present in unaffected members. Consequently, 44, 10, and 91 variants were identified in Family 1, 2, and 3, respectively (S5 Table). Further searching within the families for variants in the same gene on chromosome 3p22 showed that all three families harbored a variant in the same codon of the *SCN11A* gene: R222H (Family 1 and 2) and R222S (Family 3) (Fig 3). No other variant was shared among the three families. We found no alterations in either *SCN9A* or *SCN10A*.

To determine segregation and confirm our exome analysis, direct sequencing of *SCN11A* was performed in all six families. We found complete segregation with the episodic pain phenotype of R222H in Family 1, 2, 4, 5, and 6, and R222S in Family 3 (Fig 1).

To investigate the MAF, we genotyped *SCN11A* R222H and R222S in 105 in-house Japanese controls by RFLP. Carriers of these two variants were not found in these controls.

**Fig 4:**
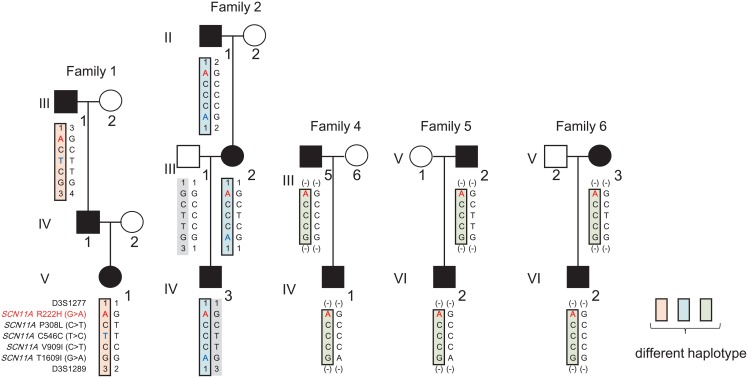
Three different haplotypes carrying R222H in five families. Haplotypes in Family 1, 2, 4, 5, and 6 are shown for five *SCN11A* variants (R222H, P308L, C546C, V909I, and T1609I) and two microsatellite markers flanking *SCN11A* (D3S1277 [Position: 34,614,226 bp, NCBI build 38.2] and D3S1289 [Position: 54,445,451 bp, NCBI build 38.2]; determined in Family 1 and 2). Variant genotypes were determined by exome analysis or direct sequencing. Microsatellite marker genotypes were determined using the ABI Prism Linkage Mapping Set. Three different haplotypes carrying R222H are represented in orange, blue, and green boxes. Red characters show the minor R222H allele. Blue characters show genotypes not shared by the other families.

Because five (Family 1, 2, 4, 5, and 6) out of six families harbor the same mutation i.e., R222H, we compared the haplotypes in these families using SNPs. Three different haplotypes were identified, indicating that R222H is derived either from a founder mutation or a private mutation (Fig 4).

### Behavioral experiments using the R222S mouse

**Fig 5:**
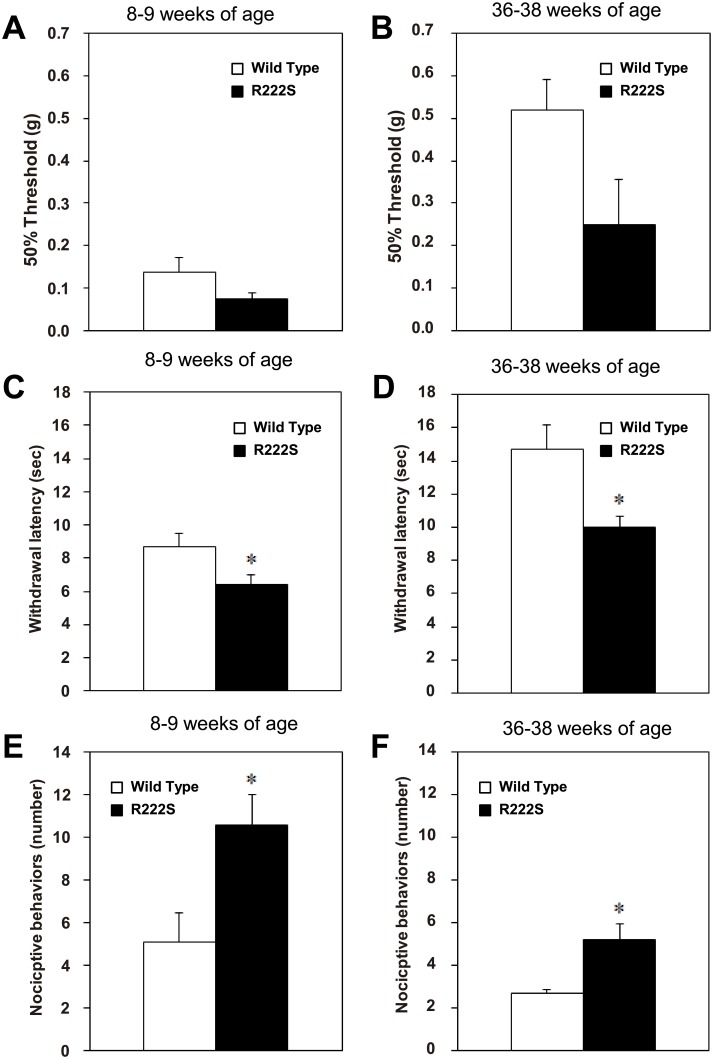
Behavioral phenotype comparison between R222S and WT mice. Sensitivity to a mechanical stimulus (determined by measuring the 50% threshold in the von Frey test) in 8–9-week-old mice (A) and 36–38-week-old mice (B); to a thermal stimulus (determined by measuring paw withdrawal latency in the Hargreaves test) in 8–9-week-old mice (C) and 36–38-week-old mice (D); to a cold stimulus (determined by measuring the number of nociceptive behaviors in the cold plate test) in 8–9-week-old mice (E) and 36–38-week-old mice (F). Open and closed columns show WT (8–9 weeks old, *n* = 10; 36–38 weeks old, *n* = 6) and R222S mice (8–9 weeks old, *n* = 12; 36–38 weeks old, *n* = 5), respectively. Data are presented as mean and S.E.M. (*p < 0.05; two-sided Student’s *t* test).

At 8–9 weeks, no abnormal signs were observed in general behavior or neurobehavioral function in R222S mice compared with WT mice (Irwin’s test). Two different age groups, 8–9-week-old and 36–38-week-old mice were used in the following behavioral experiments. Locomotor activity was measured. No significant differences between R222S and WT mice were found in either age group (S1 Fig). We compared sensitivity to mechanical, thermal, and cold stimuli between R222S and WT mice. Sensitivity to a mechanical stimulus was determined by measuring the 50% threshold in the von Frey test. In both age groups, the mean threshold value in R222S mice (8–9 weeks old, 0.07 ± 0.01 g, *n* = 12; 36–38 weeks old, 0.25 ± 0.11 g, *n* = 5) was lower than in WT mice (8–9 weeks old, 0.14 ± 0.03 g, *n* = 10; 36–38 weeks old, 0.52 ± 0.07 g, *n* = 6), but not statistically different (8–9 weeks old, *p* = 0.075; 36–38 weeks old, *p* = 0.058) (Fig 5A and 5B). Sensitivity to a thermal stimulus was determined by measuring paw withdrawal latency in the Hargreaves test. In both age groups, mean latency in R222S mice (8–9 weeks old, 6.42 ± 0.57 s, *n* = 12; 36–38 weeks old, 9.96 ± 0.68 s, *n* = 5) was significantly shorter than in WT mice (8–9 weeks old, 8.69 ± 0.80 s, *n* = 10; 36–38 weeks old, 14.71 ± 1.48 s, *n* = 6) (8–9 weeks old, *p* = 0.029; 36–38 weeks old, *p* = 0.024) (Fig 5C and 5D), indicating that R222S mice show thermal hypersensitivity compared with WT mice. Sensitivity to a cold stimulus was determined by measuring nociceptive behavior in the cold plate test. In both age groups, the number of nociceptive behaviors in R222S mice (8–9 weeks old, 10.58 ± 1.45, *n* = 12; 36–38 weeks old, 5.20 ± 0.73, *n* = 5) was significantly higher than in WT mice (8–9 weeks old, 5.10 ± 1.36, *n* = 10; 36–38 weeks old, 2.67 ± 0.21, *n* = 6) (8–9 weeks old, *p* = 0.013; 36–38 weeks old, *p* = 0.0057) (Fig 5E and 5F), indicating that R222S mice show cold hypersensitivity compared with WT mice.

### Current-clamp characterization of DRG neurons in R222S and WT mice

**Fig 6:**
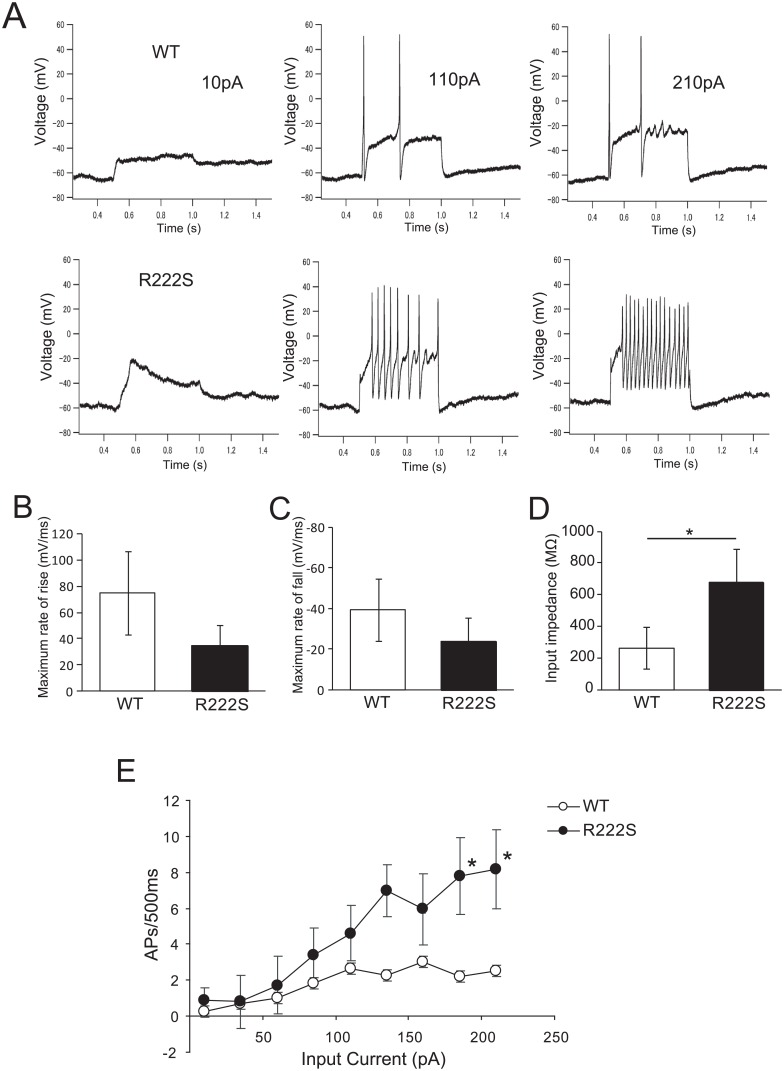
R222S mutation increases excitability in DRG neurons. (A) Small DRG neuron (< 25μm) responses to 500-ms depolarizing current steps of 10, 110, and 210 pA in WT and R222S mice. The parameter of the first AP obtained during current injections of 210 pA, showing calculated maximum rate of rise (B) and fall (C) of AP firing. Open and closed circles represent WT (*n* = 4) and R222S mice (*n* = 5), respectively. (D) Input impedance was measured at an injection current of 10 pA. Open and closed columns represent WT (*n* = 6) and R222S mice (*n* = 5), respectively. (E) Comparison of repetitive action potentials between WT and R222S mice. Open and closed circles represent WT (*n* = 6) and R222S mice (*n* = 5), respectively. The range of 500-ms-step current injections was 10–210 pA. Data are presented as mean ± S.E.M. (*p < 0.05; two-sided Student’s *t* test).

To assess the effect of the R222S mutation on DRG neuronal excitability, we performed current-clump recordings in small DRG neurons (< 25 μm) from R222S and WT mice (Fig 6). We excluded the population of neurons that did not generate APs or had only one AP firing from our analysis, since > 80% of DRG neurons that generated APs had repetitive APs.

There was no significant difference in RMP of DRG neurons between WT and R222S mice (WT, −56.17 ± 9.64 mV, *n* = 3; R222S, −51.92 ± 5.74 mV, *n* = 4; *p* = 0.54). In contrast, the amplitude of the first APs was significantly different between WT and R222S mice when measured using a current injection of 210 pA (WT, 48.17 ± 26.76 mV, *n* = 5; R222S, 26.16 ± 12.51 mV, *n* = 5; *p* < 0.05), while 50% AP width was not significantly different (WT, 5.35 ± 2.24 ms, *n* = 5; R222S, 7.41 ± 4.32 ms, *n* = 5; *p* = 0.38). The maximum rate of rise of AP (WT, 74.61 ± 31.78 mV/ms, *n* = 5; R222S, 34.69 ± 15.88 mV/ms, *n* = 5; *p* = 0.08) and fall of AP (WT, −39.26 ± 15.25 mV/ms, *n* = 5; R222S, −24.06 ± 11.29 mV/ms, *n* = 5; *p* = 0.15) was not significantly different (Fig 6B and 6C), while input impedance in R222S mice were significantly higher than in WT mice (WT, 262.0 ± 118.57 MΩ, *n* = 6; R222S, 677.43 ± 209.44 MΩ, *n* = 5; *p* < 0.05, Fig 6D). These results suggest that DRG neurons of R222S mice can fire APs even with a low stimulus input. Next, we examined firing frequency in WT and R222S mice by current injection. Representative responses for DRG neurons from R222S and WT mice are shown (Fig 6E). In R222S mice, the firing frequency increased with current injection, while firing frequency remained at a low level in WT mice. Accordingly, firing frequency (calculated from the average number of APs in 500 ms) was significantly higher (using a 185 pA input current) in R222S mice than WT mice (Fig 6E). These results suggest that excitability is higher in R222S mice than WT mice.

### Voltage-clamp characterization of Nav1.9-overexpressing ND7/23 cells

**Fig 7:**
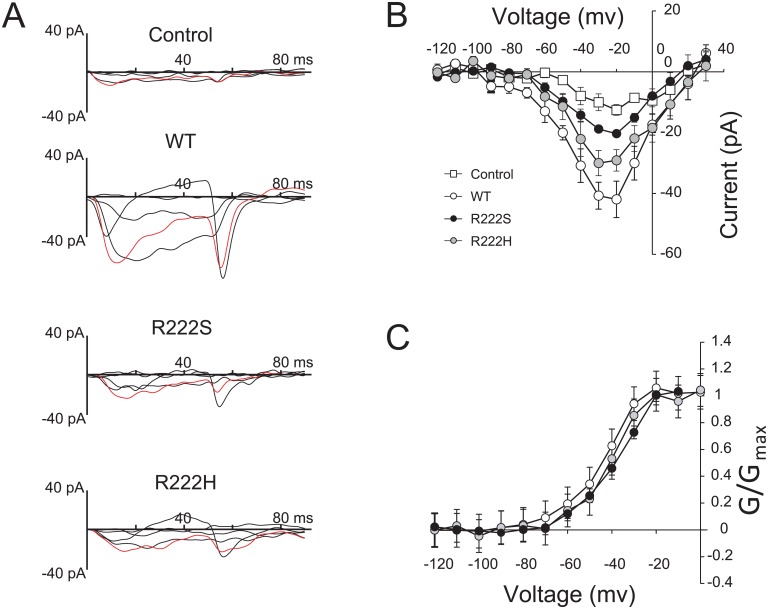
Characteristics of Nav1.9-overexpressing ND7/23 cells. (A) The typical traces of Na^+^ currents under 3 μM TTX treatment among Nav1.9-overexpressing ND7/23 cells (Control, WT, R222S, and R222H) were selected at step pulses from −80–0 mV for 100 ms with 20-mV increments for clarity. Control indicates ND7/23 cells without Nav1.9 transfection. The data were obtained at 28°C. Red-colored traces were obtained at −20 mV step pulse. (B) Current-voltage relationships for each Nav1.9-overexpressing ND7/23 cell (Control *n* = 5, WT *n* = 5, R222S *n* = 5, and R222H *n* = 4). Step pulses were applied from −120–30 mV for 100 ms in 10-mV increments. (C) Comparison of activation of each Nav1.9-overexpressing ND7/23 cell. The Boltzmann fit correspond to V_1/2_ (WT: −44.83 ± 2.44 mV, *n* = 5, R222S: −39.5 ± 2.09 mV, *n* = 5, R222H: −42.22 ± 2.91 mV, *n* = 4).

To determine the electrophysiological properties of Nav1.9 mutants (R222S and R222H), we performed voltage-clamp recordings in Nav1.9 (WT, R222S, and R222H)-overexpressing ND7/23 cells in the presence of 3 μM TTX (Fig 7A). Immunocytochemistry using anti-FLAG tag confirmed Nav1.9 expression in the plasma membrane of Nav1.9-overexpressing ND7/23 cells (S2A Fig). The peak amplitude was larger in Nav1.9 (WT)-overexpressing ND7/23 cells than control and Nav1.9 (R222S and R222H)-overexpressing ND7/23 cells (Fig 7B).

To determine the effect of gene transfer efficiency on current amplitude, we investigated the relationship between current amplitude and expression level of the WT construct. As shown (S2B Fig), current amplitudes were independent of expression levels.

The voltage-dependence of activation in Nav1.9-overexpressing ND7/23 cells was not significantly different in Nav1.9 (WT, R222S, and R222H)-overexpressing cells (mV) (V_1/2_; WT: −44.83 ± 2.44, *n* = 5, R222S: −39.5 ± 2.09, *n* = 5, R222H: −42.22 ± 2.91, *n* = 4, Fig 7C). However, we were unable to analyze the inactivation curve because these currents were so small. These results suggest that the characteristic electrophysiological properties of each mutation are not different between Nav1.9 (WT, R222S, and R222H)-overexpressing ND7/23 cells.

## Discussion

Here, we identified patients with typical childhood episodic pain symptoms in six unrelated families from throughout Japan, and further, identified two missense mutations in *SCN11A* (R222H and R222S) as the cause of the episodic pain. Given that these Nav1.9 variants are both gain-of-function mutations, the phenotypes elicited by the mutations are divergent. There are three subtypes of clinical manifestations i.e., loss of pain, painful small fiber neuropathy, and familial episodic pain (Table 1) in affected individuals with Nav1.9 variants (described in previous reports [10–12, 27, 28]). While L811P is associated with loss-of-pain phenotypes [10], other variants (e.g., G699R, L1158P, and I381T) are associated with small fiber neuropathy. These variants cause painful small fiber neuropathy of late onset i.e., fifth or sixth decades, and are complicated by skin discoloration and burning sensation, and autonomic dysfunction including a sensation of dry eyes, diarrhea, urinary problems, palpitations, and orthostatic dizziness [12, 27]. The third phenotype is familial episodic pain. The Nav1.9 mutations, R225C, A808G, and V1184A have previously been reported in two Chinese families [11] and a family of mixed European ancestry [28]. These affected cases and our affected subjects share similar clinical characteristics (Table 1): early infantile onset of episodic pain and gradual amelioration with growth, primary localization of pain in the lower limbs, induction by cold exposure or weather changes, and alleviation of pain with anti-inflammatory medication. In some cases, it may be misdiagnosed with other diseases such as growing pains, because of gradual amelioration in adolescence and no abnormal laboratory findings. Clinical severity and symptoms are diverse, even in the same family, suggesting that the phenotype is modified by non-genetic factors. Further evaluation of the genotype-phenotype correlation is needed.

**Table 1:** Three subtypes of clinical manifestations.

	Loss of pain perception	Painful small fiber neuropathy		Familial episodic pain		
References	Leipold et al ^[^^10^^]^ (2013)	Huang et al^[^^12^^]^(2014)	Han et al ^[^^27^^]^ (2015)	Zhang et al^[^^11^^]^ (2013)	Leipold et al^[^^28^^]^ (2015)	This study
**Mutation types**	L811P	L1158P and I381T	G699R	R225C and A808G	V1184A	R222H and R222S
**Number of pedigrees (or patients)**	2 families	4 patients	1 family	2 families	1 family	6 families
**Onset of pain**	NR	fifth or sixth decades	64 years old	1 year old	Within 1 or 1.5 year	Infancy
**Location of pain**	(Lip, nose, knee)	Principally distal extremities (feet, hands, toes, foot soles, fingers, ear and tip of the tongue, arms, and legs)	Feet, and hands	Principally to the lower extremities. In a few adult patients, pain located at palms, wrist, soles and knees	Lower extremities, occasionally upper. Start in joints and radiates to arms and legs	Knees, ankles, wrists and elbows in frequency order. Occasionally localized to forearms, brachium, palm, fingers, thigh, and acrotarsium
**Triggers of pain**	NR	Low ambient temperature in one patient	Warmth, exercise	Rainy days, weakness, fatigue, illness	Gluten, cold temperature, exhaustion, illness	Weather change, cold temperature, fatigue
**Frequency of pain**	NR	NR	NR	6–8 times/month	2–3 times/month	about 10 times/month (unstable)
**Duration of pain**	NR	NR	NR	15–20 min	20–30 min	15–30 min (~2 hours)
**Response to medication**	NR	Gabapentin, acetaminophen, arthrotec, nortriptyline	NR	Ibuprofen	Ibuprofen, Naproxen, Colchicine	Acetaminophen, Ibuprofen, Loxoprofen
**Painful time of the day**	NR	Tend to be severe in the morning	NR	Late in the day	Late afternoon, early evening or night	Occasionally occur during the night, while asleep
**Cold feeling**	NR	NR	NR	Yes	NR	Yes
**Burning sensation**	NR	Yes	Yes	NR	Yes	No
**Autonomic symptoms**	Hyperhidrosis, gastrointestinal dysfunction	Hyperhidrosis, hot flush, dry eyes, diarrhea, urinary problems, palpitations, orthostatic dizziness	Hyperhidrosis, orthostatic dizziness, palpitations, erectile dysfunction	Sweating	Constipation (since the age of 18), diarrhea	Anorexia and diarrhea following the cease of a series of pain in a few patients
**Skin changes**	NR	Discoloration of skin, decrease of vibration sense	Redness of the skin	NR	Flushing of the neck and face	No
**Measures for pain relief**	NR	Cold breeze	Cold water or packs	Hot compress, pressure	Warmth, gluten-free diet	Warmth, massage, compress
**Age-related changes of pain**	NR	NR	NR	Decreased with age	Decreased with age	Decreased with age (since around the age of 15)
**Development**	Delayed motor development, muscle weakness. No intellectual disability	NR	NR	Normal	Normal	Normal

Clinical characteristics correspond with mutations of Nav1.9. NR was not described in these reports. Yes/No indicates the presence of symptoms.

In our study, all patients had mutations at the arginine amino acid in position 222. Haplotype analysis revealed three different haplotypes carrying R222H, indicating that R222H is derived not only from a founder mutation, but also as an independent mutation. Another mutation, R222S, also occurred at the same position. Such a coincidental amino acid position with a childhood-restricted episode strongly suggests that gain-of-function *SCN11A* mutations and a painful childhood syndrome may have been overlooked in Japan because of self-limited mitigation of pain episodes by growth. We are tempted to speculate that our cases represent only the tip of the iceberg of such infantile patients.

Our behavioral experiments using knock-in mice successfully replicated the characteristic features of painful behavior i.e., elevated cold sensitivity in patients. Furthermore, our *in vivo* study confirmed that R222S is the gain-of-function mutation, with up-regulated excitability of small DRG neurons observed in R222S knock-in mice by electrophysiological experiments.

Correspondingly, R222S mice demonstrate a chronic pain phenotype similar to that in human. This chronic pain phenotype is explained by increased DRG neuronal excitability in juvenile mice. It suggests that a mechanism of this pain is involved in higher electrical activity, thereby promoting AP firing in DRG neurons. Interestingly, the symptoms in these mice did not disappear after growth, which is different from the human case. A previous study has shown that Nav1.9 glycosylation state is paralleled by developmental change. Moreover, enzymatic desialidation of neonatal DRG cultures change TTXr properties in rat DRG cells [29]. However, in the present study, the mouse R222S knock-in model did not replicate growth-dependent alleviation of pain sensitivity, suggesting that the rodent model does not represent age dependency exactly as in human familial episodic pain syndrome. Thermal hypersensitivity in R222S mice also differed from the finding that patients ameliorated their pain by warming the lesion. Further investigation may reveal the correlation between cold/thermal sensitivity and Nav1.9 function.

Recent studies in humans have reported that 12 amino acid substitutions of Nav1.9 are relevant to pain disorders, with seven of them associated with gain-of-function and painful/painless phenotypes [10–12, 27, 28] (Table 1). Particularly, in a Chinese family with autosomal-dominant episodic pain, a point mutation of *SCN11A* (R225C) alters the firing property despite no significant difference in RMP and threshold [11]. This mutation is positioned at the S4 segment, which is the voltage censor domain. A previous study has shown that the S4 region in all four domains of rat brain IIA and II sodium channels are involved in both activation and inactivation gating [30, 31]. Therefore, the R225C mutation was expected to change voltage-dependent gating of ion channels. However, G_max_ and I_max_ does not change significantly [11]. In mice, the R222S mutation is also in one of the multiple repeats of a motif consisting of a positively charged residue in the S4 segment, and did not change RMP by current-clamp recording. We attempted to confirm the gating properties of the Nav1.9 mutations, R222S and R222H. Activation curves from Nav1.9 mutation-overexpressing ND7/23 cells were not significantly different with voltage steps. Our results are in accordance with observations on the R225C mutation, which is located near this mutation [11]. However, inactivation gating was not confirmed in Nav1.9-overexpressing ND7/23 cells because these currents were so small. A lack of inactivation data is one limitation of our current study. Thus, understanding of the detailed mechanism underlying the gating property still requires further investigation.

Nav1.7, 1.8, and 1.9 are preferentially expressed in peripheral neurons and are implicated in injury-induced neuronal hyperexcitability [11, 32, 33]. Recent studies have revealed an interaction between a scaffolding protein and TTXs/TTXr neuronal channels. The binding domains are weakly conserved and the core motif of the domain shared between potassium channels [34, 35]. Particularly, Nav1.8 interacts constitutively with ankyrin G in skin nerve fibers. This constitutive binding may contribute to pathological aspects of the illnesses caused by Nav1.8 ectopic expression [36, 37]. We demonstrated that the amplitude of the first AP was changed by R222S mutation. The ratio of TTXs/TTXr currents will affect excitability [38]. Additionally, Kv1 expression is reported in small DRG neurons [39]. Kv1 channels control the firing properties of neurons to generate a single spike at the onset of stimulus, and application of DTX, a selective potassium channel blocker, blocks Kv1 channels and induces repetitive firing in the auditory system [40, 41]. We found that the R222S mutation altered input resistance but did not significantly affect the maximum rate of rise/fall of AP. These results are suggestive of a mechanism whereby the R222S mutation may change the interplay with axonal channel components in AP firing, including channel localization and distribution.

Impaired Nav1.9 function is correlated with three types of neuronal phenotypes: painless, small fiber neuropathy, and familial episodic pain syndrome (Table 1). Onset of the latter two phenotypes seems to be highly age dependent, but with the age of onset reversed: elderly for small fiber neuropathy and infancy for familial episodic pain syndrome. Genotype and phenotype association has never been explored for Nav1.9 age dependency. A previous study has shown that Dravet Syndrome, with progressive infantile onset epileptic encephalopathy caused by loss-of-function mutations in *SCN1A*, is correlated with natural loss of Nav1.3 channel expression in brain development, coupled with failure of increased functional Nav 1.1 channels [42]. Expression of TTXs and TTXr is regulated by developmental changes of neurotropic factors and/or a shift in expression of specific neurotrophic receptors [43]. Thus, further studies are warranted to elucidate the mechanisms of such an age dependency.

## Conclusions

In the present study, we identified *SCN11A* R222H and R222S in six unrelated Japanese families with familial episodic pain syndrome. For R222S, we show the pathological role of this mutation in familial episodic pain syndrome using a mouse knock-in model combined with behavioral and electrophysiological examinations. The mouse model developed here will be useful for drug screening for familial episodic pain syndrome associated with *SCN11A*.

## Supporting Information

S1 Fig Locomotor activity in different age groups. (PDF)

S2 Fig Expression of Nav1.9-3x FLAG (wild-type, R222S, and R222H) in ND7/23 cells. (A) Nav1.9-3xFLAG immunoreactivity is shown in red, with positive reactivity in Nav1.9-overexpressing ND7/23 cells at the membrane surface. Scale bar: 10 μm. (B) Expression levels of Nav1.9-3xFLAG (wild-type [WT], R222S, and R222H) in ND7/23 cells. Low WT indicates another Nav1.9 (WT)-overexpressing ND7/23 cell clone with lower expression. Expression levels were not significantly different in WT, R222S, and R222H cells (*p* > 0.38). (C) There was no significant difference in current amplitude and voltage dependency in expression levels of Nav1.9-3x FLAG (low WT; *n* = 4, WT; *n* = 5). Control: ND7/23 cells without Nav1.9 transfection.
(PDF)

S1 Table Primers used for *SCN11A* amplification. (DOCX)

S2 Table Primers and enzymes for RFLP methods. (DOCX)

S3 Table Oligonucleotides and primers for Nav1.9 knock-in mouse production. (DOCX)

S4 Table Primers used for real-time quantitative PCR of SCN11A cDNA. (DOCX)

S5 Table Number of variants and candidate genes for each family. (DOCX)
