# Supplementary figures and images for: Infantile Pain Episodes Associated with Novel Nav1.9 Mutations in Familial Episodic Pain Syndrome in Japanese Families
Source: PLoS One. 2016 May 25;11(5):e0154827. doi: 10.1371/journal.pone.0154827 (PMC4880298; doi:10.1371/journal.pone.0154827)

# Locomotor activity

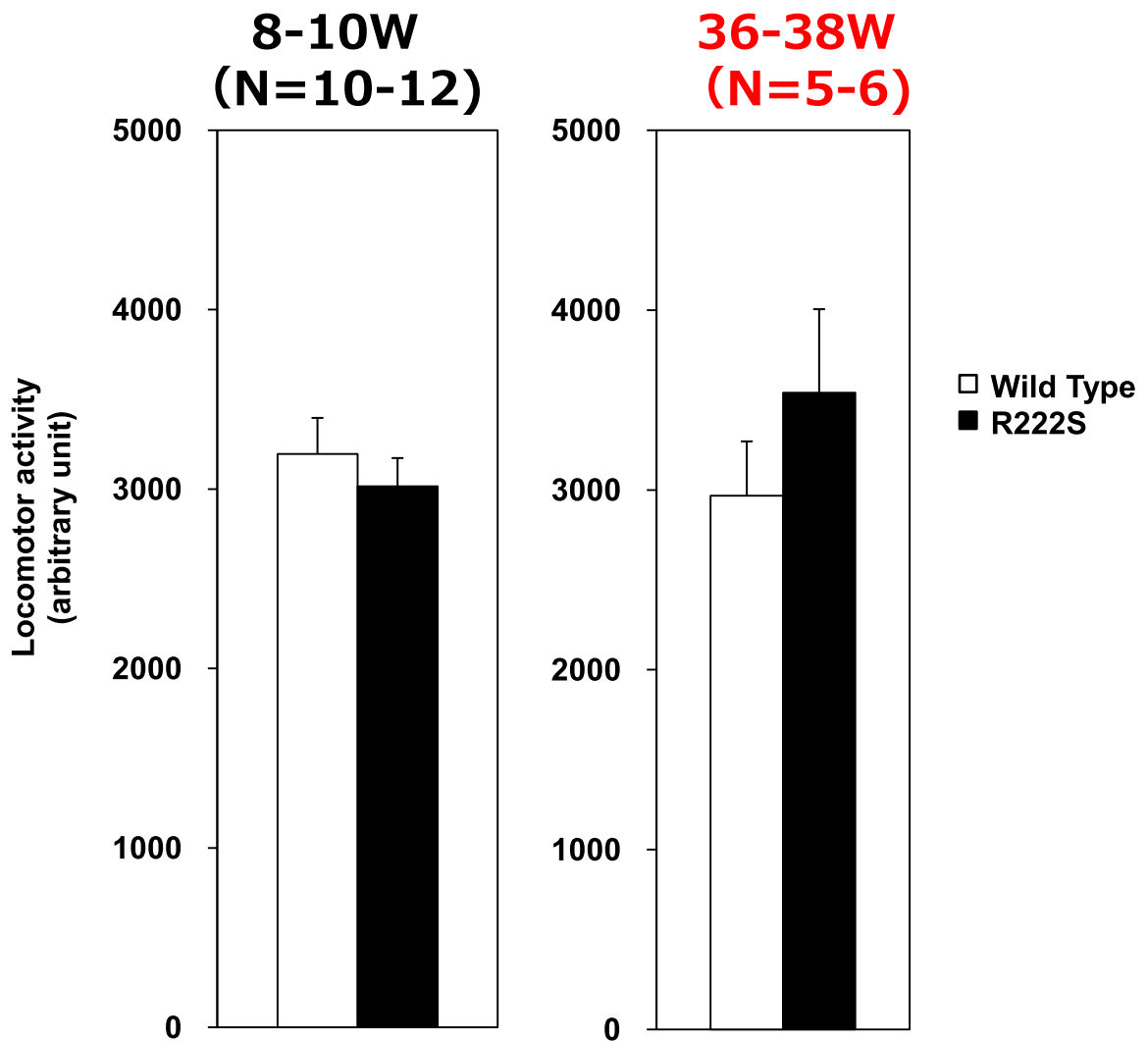

**S1 Fig** Locomotor activity in different age groups.

Supplement: S1 Fig — (PDF) [file pone.0154827.s001.pdf]
