# Supplementary material for: Infantile Pain Episodes Associated with Novel Nav1.9 Mutations in Familial Episodic Pain Syndrome in Japanese Families
Source: PLoS One. 2016 May 25;11(5):e0154827. doi: 10.1371/journal.pone.0154827 (PMC4880298; doi:10.1371/journal.pone.0154827)
Supplement: S2 Fig — (A) Nav1.9-3xFLAG immunoreactivity is shown in red, with positive reactivity in Nav1.9-overexpressing ND7/23 cells at the membrane surface. Scale bar: 10 μm. (B) Expression levels of Nav1.9-3xFLAG (wild-type [WT], R222S, and R222H) in ND7/23 cells. Low WT indicates another Nav1.9 (WT)-overexpressing ND7/23 cell clone with lower expression. Expression levels were not significantly different in WT, R222S, and R222H cells (p > 0.38). (C) There was no significant difference in current amplitude and voltage dependency in expression levels of Nav1.9-3x FLAG (low WT; n = 4, WT; n = 5). Control: ND7/23 cells without Nav1.9 transfection. (PDF) [file pone.0154827.s002.pdf]

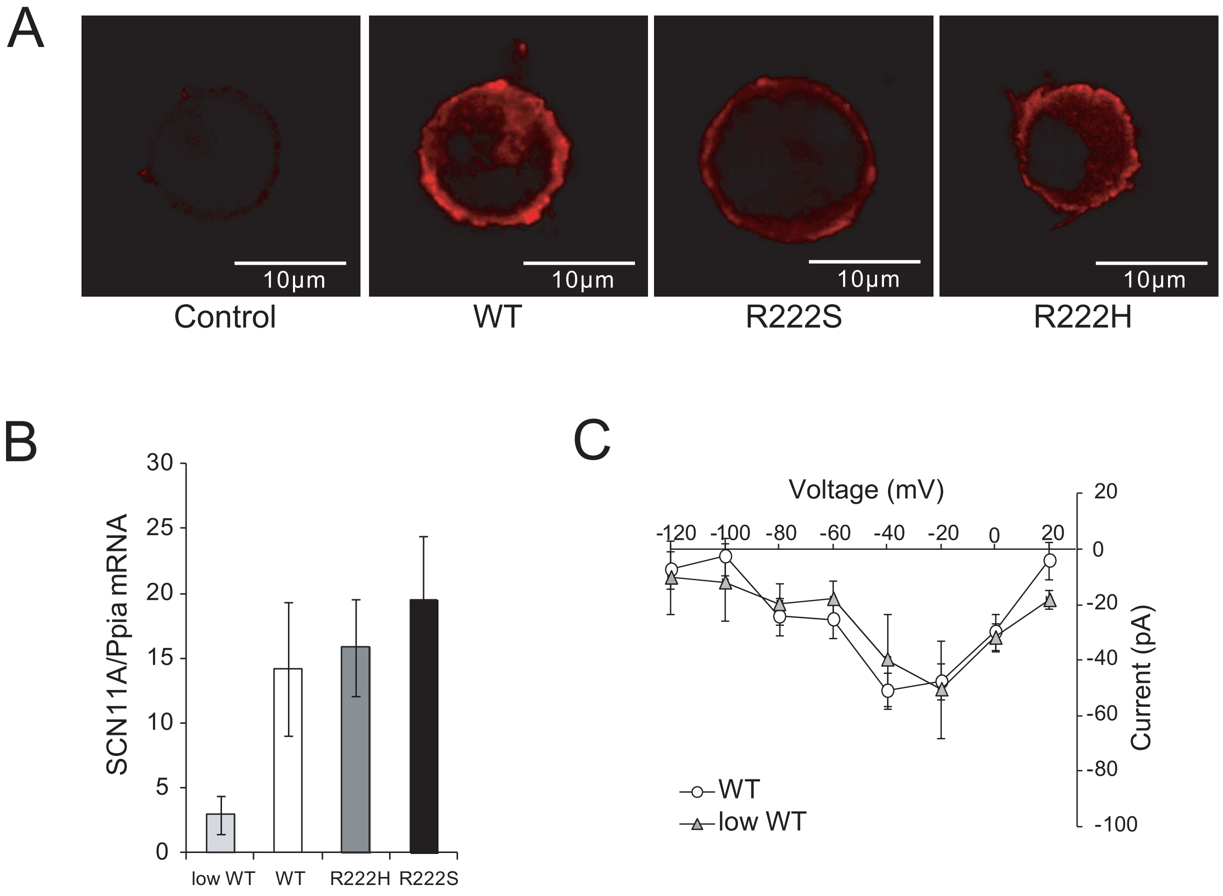

S2 Fig Expression of Nav1.9-3x FLAG (wild-type, R222S, and R222H) in ND7/23 cells.

(A) Nav1.9-3xFLAG immunoreactivity is shown in red, with positive reactivity in Nav1.9-overexpressing ND7/23 cells at the membrane surface. Scale bar: 10 μm. (B) Expression levels of Nav1.9-3xFLAG (wild-type [WT], R222S, and R222H) in ND7/23 cells. Low WT indicates another Nav1.9 (WT)-overexpressing ND7/23 cell clone with lower expression. Expression levels were not significantly different in WT, R222S, and R222H cells ($p > 0.38$). (C) There was no significant difference in current amplitude and voltage dependency in expression levels of Nav1.9-3x FLAG (low WT; $n = 4$, WT; $n = 5$). Control: ND7/23 cells without Nav1.9 transfection
