# Supplementary material for: Infantile Pain Episodes Associated with Novel Nav1.9 Mutations in Familial Episodic Pain Syndrome in Japanese Families
Source: PLoS One. 2016 May 25;11(5):e0154827. doi: 10.1371/journal.pone.0154827 (PMC4880298; doi:10.1371/journal.pone.0154827)
Supplement: S1 Table — (DOCX) [file pone.0154827.s003.docx]

**S1 Table.** Primers used for amplification of *SCN11A*

| exon | Forward Primer (5' > 3') | Reverse Primer (5' > 3') |
| --- | --- | --- |
| 2 | AACTCAACTTCCCGCCTTTT | CCTGCCTGCTATAAACCCTAA |
| 3 | TTCCACAGCCCAACAGTTTAT | ATCTTGCTCACATCCAGCACA |
| 4 | TAGATGCACCTGCAGAAAAGG | GGACAGGTGAGTGAAGGAAAA |
| 5 | GGAAAAAAAAATCAAGGCCAG | TTCAGGGCCAACCAACACA |
| 6* | ACAGTGGTATTGCCAGATCCT | TCAAGCAGTTAGCACAGTGCC |
| 7 | AGCACAAACTCAAGGCATCA | GAAGCCAAAGAATGAGGCAA |
| 8 | TCTTGGGCTCCAGTTTCTCTC | TGCCTTAAACACCAGGAAATG |
| 9 | ACAGTGCAGTGGGCCACTTTA | AAAAGTGGGGGATGAATGGT |
| 10 | GGGAAGCTATATGGTCTGTGG | TGAAAGATAGCAGCACAGCCA |
| 11 | CAGAGCTAGCGTAGTCCAGGT | AGACACATGGATGCATGAAAG |
| 12 | TAAGTTTTGAGGAGAGGCAGC | TTCCAAAACAGCCTCCTTTG |
| 13 | TGGTGGAAAAACCTTTCTGAC | AACAGCCATCTTTTCCCTCA |
| 14 | TACTTCCCTTGGGCCATTCTT | TCAGTTAAGCGAAGTCCCCC |
| 15 | GCGATCTGATGGCTATGTTCA | AATTTGGGGGCATCAGTCAA |
| 16 | GCTTTCTTAGGAGACAGTGGG | CAGCCACGTTTTGTACCCTTA |
| 17 | TGTTGAGTTTTTCCAGGAGCA | AAAGTTGAGAAACCTGGCCT |
| 18 | TCATCCTAGAAACCTTTGCCT | CGACAATCGCCTCAAGAATCT |
| 19 | ACATGGCAGGGACCAGATTTA | AACCAGCAGCATCAGAGACCA |
| 20 | TTGAGCAGAACCTCATTACGA | AGACATCCATATGCGGCACA |
| 21 | GAGTGCATTGTTCCCACGTTT | CAAATTCTAAGCACTGGGCA |
| 22 | ATGGGGCACATGTATGTGGA | TTCCAGAAGGCATTGGCTTT |
| 23 | TCCTAGAAGGTTGGGGTATCA | AAACAAGGCAGAGAACCCCAG |
| 24 | TGGCTGTTAGGAAGAGGCTAT | TTTGAGGATCATCTCCAGGCA |
| 25 | TTCGCTATGGTCCAGTCTCTT | GAAGGATGGCATTACGGAAA |
| 26 | AGCCCAAGCACTTAAAACCA | AGAATGGCATTAGTACCCCTG |
| 27 | ATTGATAAAAAGTTGGCCCC | TACACTAAGCTGCTGACCCCT |

*p.R222H/S is located exon 6 of *SCN11A*
