# Supplementary material for: Infantile Pain Episodes Associated with Novel Nav1.9 Mutations in Familial Episodic Pain Syndrome in Japanese Families
Source: PLoS One. 2016 May 25;11(5):e0154827. doi: 10.1371/journal.pone.0154827 (PMC4880298; doi:10.1371/journal.pone.0154827)
Supplement: S2 Table — (DOCX) [file pone.0154827.s004.docx]

**S2 Table.** Primers and enzymes for RFLP methods

| Mutation | Forward Primer (5' > 3') | Reverse Primer (5' > 3') | Restriction enzyme |
| --- | --- | --- | --- |
| SCN11A p.R222H | ACAGTGGTATTGCCAGATCCT | TCAAGCAGTTAGCACAGTGCC | CviQi |
| SCN11A p.R222S | ACAGTGGTATTGCCAGATCCT | TCAAGCAGTTAGCACAGTGCC | DdeI |
