# Supplementary material for: Infantile Pain Episodes Associated with Novel Nav1.9 Mutations in Familial Episodic Pain Syndrome in Japanese Families
Source: PLoS One. 2016 May 25;11(5):e0154827. doi: 10.1371/journal.pone.0154827 (PMC4880298; doi:10.1371/journal.pone.0154827)
Supplement: S3 Table — (DOCX) [file pone.0154827.s005.docx]

**S3 Table.** Oligonucleotides and primers for production of Nav1.9 knock-in mouse

| Oligonucleotides used to express sgRNA | | |
| --- | --- | --- |
| guideRNA | Oligonucleotide | Sequence |
| Scn11a-1 | Scn11a-1-sense | 5’-CAC CGC ACT CGG AAG GTA CGT AGA G-3’ |
|  | Scn11a-1-antisense | 5’-AAA CCT CTA CGT ACC TTC CGA GTG -3’ |
| Scn11a-2 | Scn11a-2-sense | 5’-CAC CGC AGA GCT CTC AAC ACT CGG A-3’ |
|  | Scn11a-2-antisense | 5’-AAA CTC CGA GTG TTG AGA GCT CTG-3’ |
|  |  |  |
| Donor oligoDNA to introduce R222S mutation | | |
| Donor oligoDNA | 5’- CCA GTG GCT TAC CTG AGA TTA CAG AAA TGG CTT TCA GAG CTC TCA ACA CTC GGA AGG TAC TTA GAG TGG AAA GAT TAT TGA CTT TGT TAC CGA GAA AAC AAG GCG CTA TCC TGG AAG ACA A-3’ | |
|  |  |  |
| Primers used for direct sequence analysis | | |
| PCR amplification | Forward | 5'-TGC ATG CTC CTG GCT ATT GAA GAT G-3` |
|  | Reverse | 5'-TCA GAA ATG TTC TAA TGC CAA CGA GC-3' |
| Sequencing | | 5'-TGG TGC CTT GTG CTT ATA ATG-3' |
