# Supplementary material for: Infantile Pain Episodes Associated with Novel Nav1.9 Mutations in Familial Episodic Pain Syndrome in Japanese Families
Source: PLoS One. 2016 May 25;11(5):e0154827. doi: 10.1371/journal.pone.0154827 (PMC4880298; doi:10.1371/journal.pone.0154827)
Supplement: S4 Table — (DOCX) [file pone.0154827.s006.docx]

**S4 Table.** Primers used for real-time quantitative PCR for *SCN11A* cDNA

| Forward Primer (5' > 3') | TCCTAGTGGTATTCCGCATCC |
| --- | --- |
| Reverse Primer (5' > 3') | TTTTGCTTCCTGCACCACTT |
