# Supplementary material for: Infantile Pain Episodes Associated with Novel Nav1.9 Mutations in Familial Episodic Pain Syndrome in Japanese Families
Source: PLoS One. 2016 May 25;11(5):e0154827. doi: 10.1371/journal.pone.0154827 (PMC4880298; doi:10.1371/journal.pone.0154827)
Supplement: S5 Table — (DOCX) [file pone.0154827.s007.docx]

**S5 Table.** Numbers of variants and candidate genes for each family

| Filtering Step | Family 1 (3 persons) | Family 2 (8 persons) | Family 3 (3 persons) |
| --- | --- | --- | --- |
| No. of variants (average) | 887223 | 1137792 | 719084 |
| Not synonymous variant | 14744 | 15306 | 13235 |
| Read depth≥8 | 13586 | 12205 | 11452 |
| Not in dbSNP135 | 643 | 604 | 435 |
| MAF<0.01 in 1K genome JPT | 584 | 551 | 388 |
| Heterozygote in affected member and not in unaffected member | 44 | 10 | 91 |
|  |  |  |  |
|  | **SCN11A (1)** | **SCN11A (1)** | **SCN11A (1)** |
|  | NOTCH2 (1) | ANKRD23 (1) | HIVEP3 (1) |
|  | TCHH (1) | SHISA5 (1) | KIAA1107 (1) |
|  | POU2F1(1) | STAB1 (1) | ZNF687 (1) |
|  | PRRC2C (1) | IKBKB (1) | FAM78B (1) |
|  | TNN (1) | RPAP1 (1) | LAMC2 (1) |
|  | PLXNA2 (1) | CAPN3 (1) | TPR (1) |
|  | PSAPL1 (1) | ZNF609 (1) | FAM58BP (1) |
|  | POU4F2 (2) | ITGA11 (1) | ROCK2 (1) |
|  | MICA (1) | CIB2 (1) | ANKRD53 (1) |
|  | LATS1(1) |  | COBLL1 (1) |
|  | ULBP2 (1) |  | PIKFYVE (1) |
|  | ARID1B (1) |  | GIGYF2 (1) |
|  | RP1L1(4) |  | C2orf85 (1) |
|  | HOOK3 (1) |  | TGM4 (1) |
|  | PPP1R16A (1) |  | ADAMTS9 (1) |
|  | TNC (1) |  | LRIG1 (1) |
|  | LCN8 (1) |  | CWH43 (1) |
|  | FAM157B (1) |  | TNPO1 (1) |
|  | C10orf93 (1) |  | GPR98 (1) |
|  | MICAL2 (1) |  | SQSTM1 (1) |
|  | DSCAML1 (1) |  | C6orf146 (1) |
|  | CXCR5 (1) |  | MAK (1) |
|  | SPATA19 (1) |  | EYS (1) |
|  | KRT2 (1) |  | FAM46A (1) |
|  | KRT4 (1) |  | AHI1 (1) |
|  | NOS1 (1) |  | TBP (1) |
|  | RIMBP2 (1) |  | HOXA3 (1) |
|  | GOLGA6L2 (1) |  | AKAP9 (1) |
|  | TCF12 (1) |  | DLX6 (1) |
|  | KIAA0430 (2) |  | PCOLCE (1) |
|  | ZNF469 (2) |  | SRRT (1) |
|  | KRTAP4-3 (1) |  | ZC3HAV1 (1) |
|  | PLIN4 (2) |  | AC021218.2 (1) |
|  | PI3 (1) |  | IDO1 (1) |
|  | NEURL2 (1) |  | TRIM55 (1) |
|  | PDE9A (1) |  | ODF1 (1) |
|  |  |  | TG (1) |
|  |  |  | KIAA1161 (1) |
|  |  |  | FAM166B (1) |
|  |  |  | FOXB2 (1) |
|  |  |  | WNK2 (1) |
|  |  |  | PKN3 (1) |
|  |  |  | FAM208B (1) |
|  |  |  | ZFAND4 (1) |
|  |  |  | PCDH15 (1) |
|  |  |  | COL13A1 (1) |
|  |  |  | FAM149B1 (2) |
|  |  |  | OPN4 (1) |
|  |  |  | BTBD16 (1) |
|  |  |  | RAPSN (1) |
|  |  |  | OR10AG1 (1) |
|  |  |  | SC5DL (1) |
|  |  |  | CELA1 (1) |
|  |  |  | MSRB3 (1) |
|  |  |  | COQ5 (1) |
|  |  |  | HNF1A (1) |
|  |  |  | ZCCHC8 (1) |
|  |  |  | SPERT (1) |
|  |  |  | SALL2 (1) |
|  |  |  | SIPA1L1 (1) |
|  |  |  | ABCD4 (1) |
|  |  |  | PPP4R4 (1) |
|  |  |  | AHNAK2 (1) |
|  |  |  | ZNF770 (1) |
|  |  |  | WDR90 (1) |
|  |  |  | DPEP2 (1) |
|  |  |  | WDR59 (1) |
|  |  |  | ATP2C2 (1) |
|  |  |  | C17orf74 (1) |
|  |  |  | MYH8 (1) |
|  |  |  | MYH4 (1) |
|  |  |  | LRRC48 (1) |
|  |  |  | TTLL6 (1) |
|  |  |  | ABCA8 (1) |
|  |  |  | NUP85 (1) |
|  |  |  | AATK (1) |
|  |  |  | ADAT3 (1) |
|  |  |  | MUC16 (1) |
|  |  |  | ZNF846 (1) |
|  |  |  | C19orf57 (1) |
|  |  |  | ZNF90 (1) |
|  |  |  | SIPA1L3 (1) |
|  |  |  | ECH1 (1) |
|  |  |  | ZNF528 (1) |
|  |  |  | KIR2DL3 (1) |
|  |  |  | PDXK (1) |
|  |  |  | TTC28 (1) |
|  |  |  | ACO2 (1) |
|  |  |  | TTLL8 (1) |
| Located in 3p22 | **SCN11A R222H** | **SCN11A R222H** | **SCN11A R222S** |

Members in each family for exome analysis were described in Figure 1A.

The numbers in the bracket indicate the number of variants for each candidate gene.
